# Type A thymoma: a rare cause of neoplastic cardiac tamponade with long-term survival

**DOI:** 10.1186/s12890-022-02034-7

**Published:** 2022-06-22

**Authors:** Mika Takashima, Kozo Kagawa, Toru Sawada, Hiroyuki Hino, Keishi Naruse, Eiji Takeuchi, Shoji Sakiyama, Tsutomu Shinohara

**Affiliations:** 1 Division of Thoracic Surgery, National Hospital Organization Kochi Hospital, 1-2-25 Asakuranishimachi, Kochi, 780-8077 Japan; 2 Department of Thoracic, Endocrine Surgery and Oncology, Graduate School of Biomedical Sciences, Tokushima University, 3-18-15 Kuramoto-cho, Tokushima, 770-8503 Japan; 3 Division of Pulmonary Medicine, National Hospital Organization Kochi Hospital, 1-2-25 Asakuranishimachi, Kochi, 780-8077 Japan; 4 Department of Respiratory Medicine and Rheumatology, Graduate School of Biomedical Sciences, Tokushima University, 3-18-15 Kuramoto-cho, Tokushima, 770-8503 Japan; 5 Division of Pathology, National Hospital Organization Kochi Hospital, 1-2-25 Asakuranishimachi, Kochi, 780-8077 Japan; 6 Department of Clinical Investigation, National Hospital Organization Kochi Hospital, 1-2-25 Asakuranishimachi, Kochi, 780-8077 Japan; 7 Department of Community Medicine for Respirology, Graduate School of Biomedical Sciences, Tokushima University, 3-18-15 Kuramoto-cho, Tokushima, 770-8503 Japan

**Keywords:** Cardiac tamponade, Type A thymoma, Multidisciplinary treatment, Long-term survival

## Abstract

**Background:**

The prognosis of thymoma with cardiac tamponade is generally poor. Most of the reported thymomas with cardiac tamponade were type B or type AB (mixed thymoma), and cardiac tamponade due to type A thymoma, which has a better prognosis compared to type B thymoma, is extremely rare.

**Case presentation:**

We encountered a case of cardiac tamponade in a 71-year-old male. He visited our emergency department due to exacerbation of fatigue and dyspnea on exertion that lasted for two weeks. Chest imaging revealed a large amount of pericardial fluid and a contrast-enhanced tumor with calcification in the anterior mediastinum. The patient underwent thoracoscopic tumor biopsy and pathological examinations revealed type A thymoma. In this case, long-term disease-free survival (7.5 years) was achieved by multidisciplinary treatment (preoperative chemotherapy, surgical excision, and postoperative radiation therapy), in accordance with the histological type.

**Conclusions:**

This case indicates that neoplastic cardiac tamponade, even in elderly patients, should not necessarily be regarded as a terminal cancer and requires a systematic investigation for underlying causes.

## Background

Thymoma is the most common primary neoplasm among anterior mediastinal lesions and usually asymptomatic. However, thymoma should be considered in the differential diagnosis of cardiac tamponade as the initial symptom of latent disease [1, 2]. The prognosis of thymoma depends on the staging and histological classification. In general, type A thymoma has a better prognosis compared to type B thymoma but there are few reports on the prognosis of type A thymoma with cardiac tamponade [3, 4]. Here, we report a case of cardiac tamponade due to type A thymoma in an elderly patient in which long-term disease-free survival was achieved by multidisciplinary treatment.

## Case presentation

**Fig. 1:**
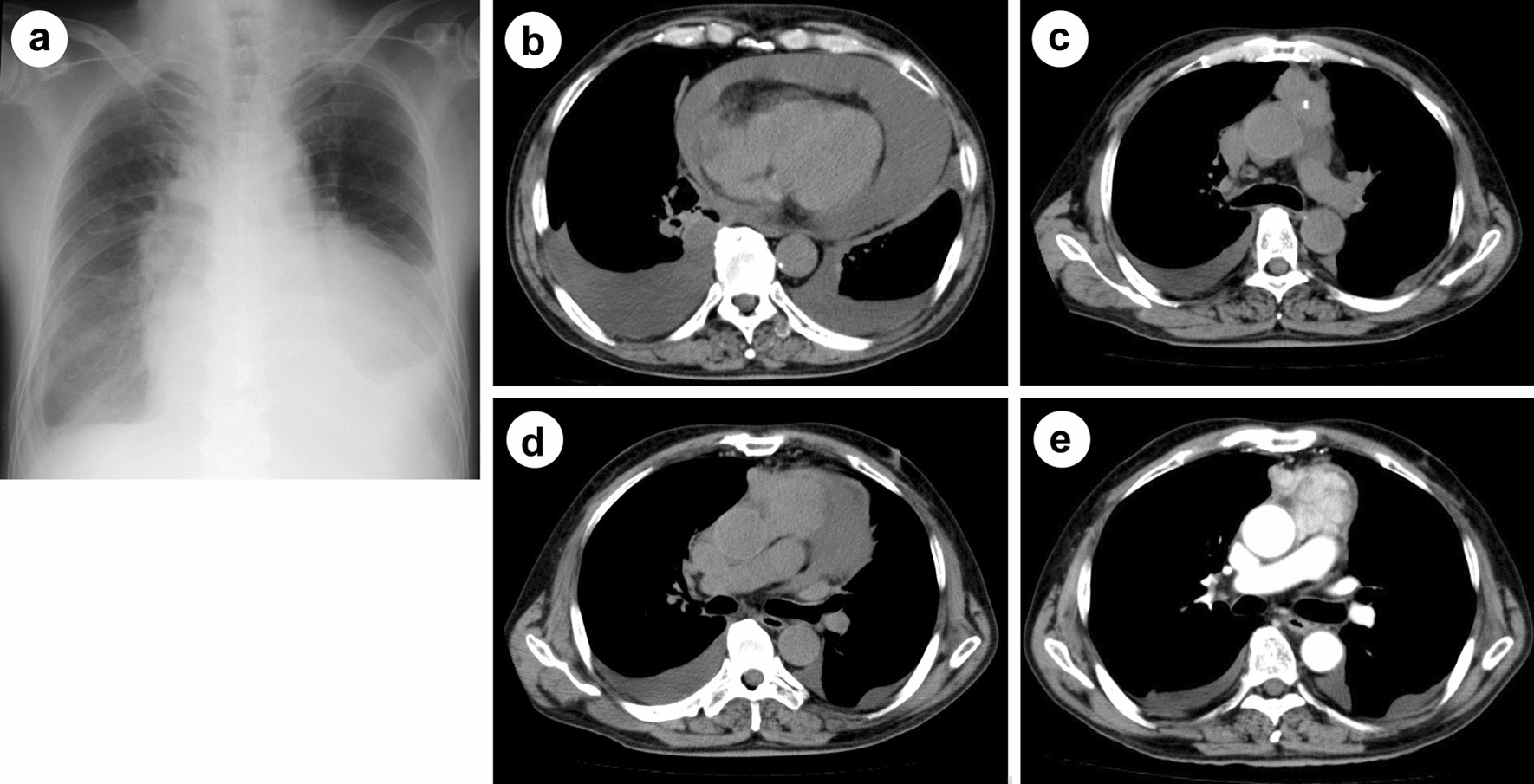
Radiological findings. **a**, **b** A large amount of pericardial fluid and bilateral pleural effusion. **c**, **d** A tumor with calcification in the anterior mediastinum extending into the pericardial inner lumen. **e** A contrast-enhanced tumor in the anterior mediastinum during drainage

**Fig. 2:**
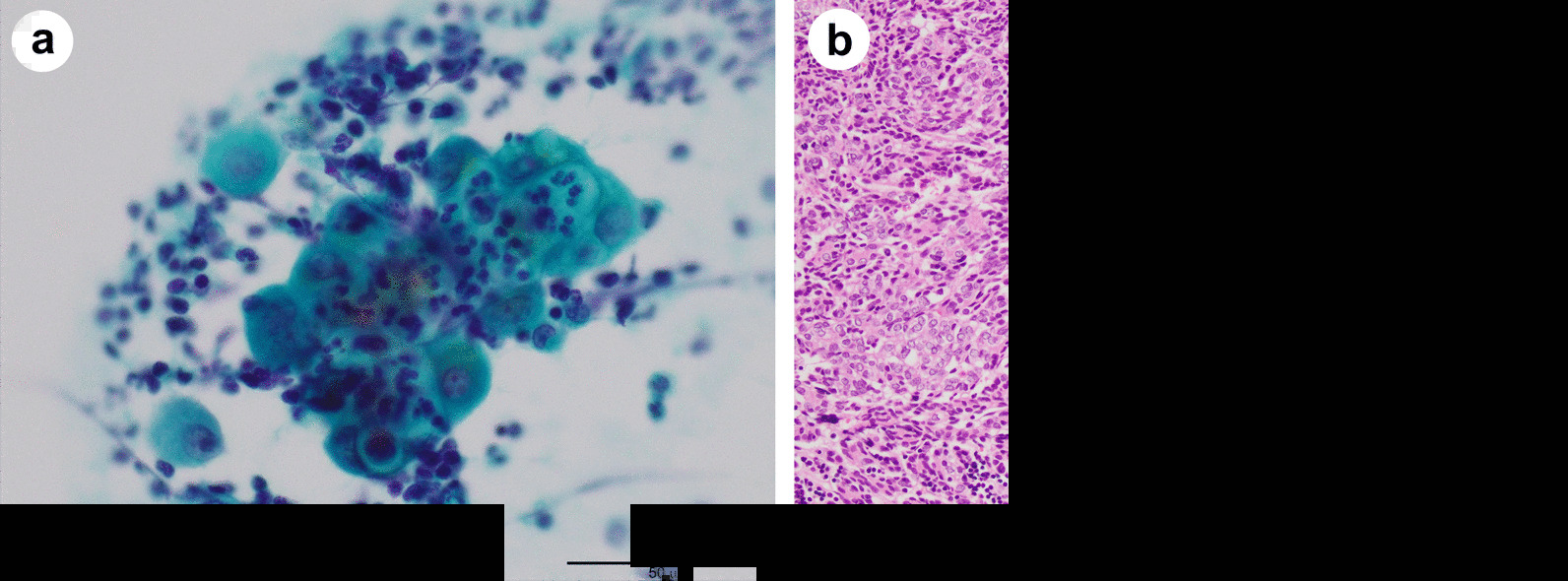
Cytology of pericardial fluid (Papanicolaou stain, × 400) (**a**) and histology of a biopsy specimen (Hematoxylin and eosin stain, × 200) (**b**). **a** Pericardial fluid showing an aggregate of atypical cells and inflammatory cells (mainly neutrophils). Atypical cells contain large nuclei with poor chromatin enrichment, relatively prominent nucleoli and abundant cytoplasm. **b** Spindle-shaped and oval epithelial cells with low-grade dysplasia arranged in pseudo-rosettes or short fascicles admixed with few lymphocytes. The sections were observed with a microscope: BX53 (OLYMPUS, Tokyo, Japan), lenses: UPlanFl (OLYMPUS, Tokyo, Japan), a camera: DP22 (OLYMPUS, Tokyo Japan), and a photo system: cellSens Standard 2.3 (OLYMPUS, Tokyo Japan) at a resolution of 96 dots per inch (1920 × 1440 pixels). The scale bar is 50 μm (**a**) or 100 μm (**b**)

**Fig. 3:**
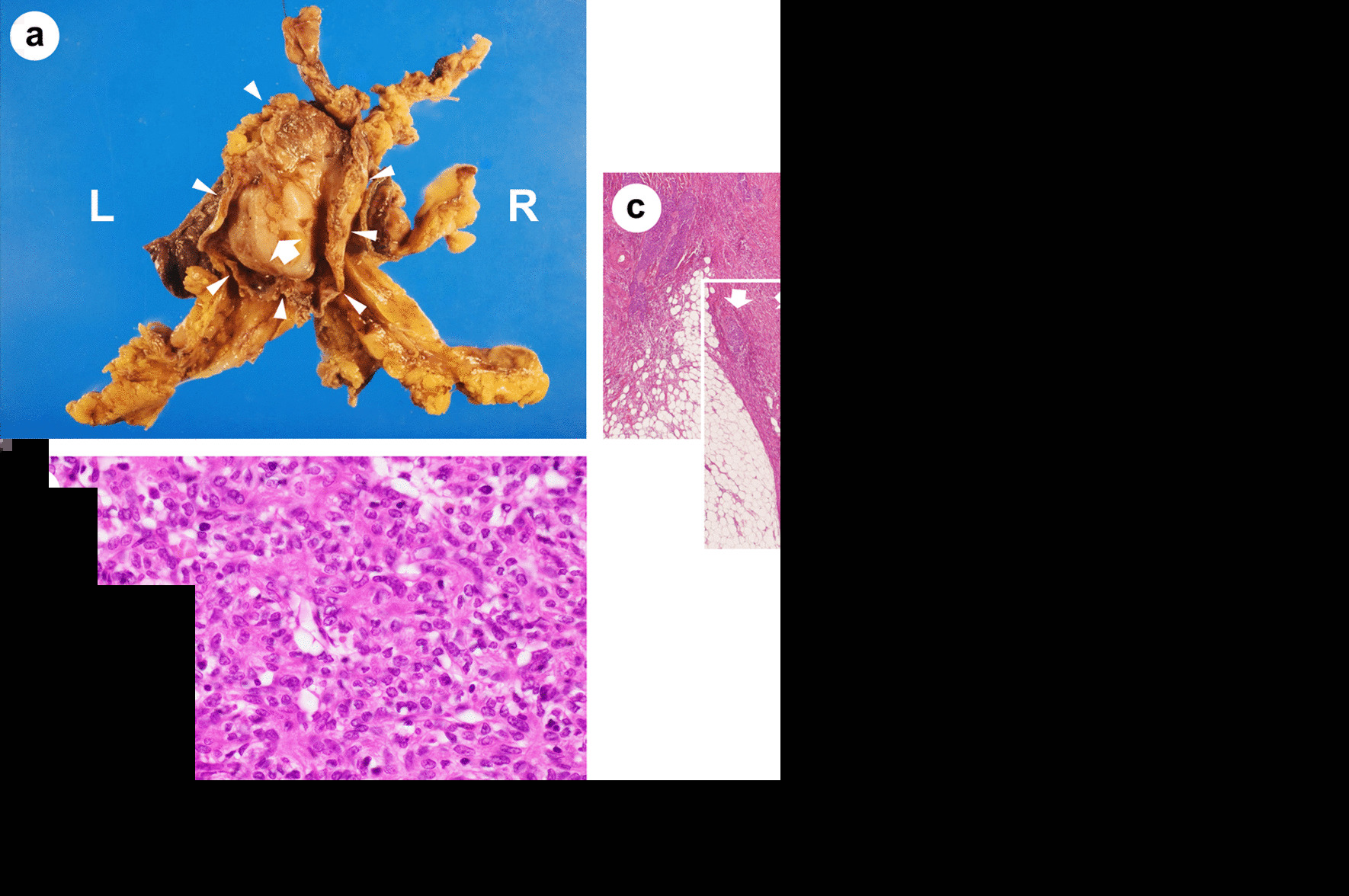
Dorsal view of a formalin-fixed resected specimen (**a**) and histology (**b–e**) (Hematoxylin and eosin stain: **b**, × 400; **c–e**, × 40). **a** A tumor (white arrow) extending into the pericardial inner lumen (white arrowhead: pericardium). **b** Type A thymoma showing the same morphology as at the time of biopsy. **c–e** Tumor cell invasion into the pericardium (white arrow). **b** Microscope: BX53 (OLYMPUS, Tokyo, Japan), lenses: UPlanFl (OLYMPUS, Tokyo, Japan), a camera: DP22 (OLYMPUS, Tokyo Japan), and a photo system: cellSens Standard 2.3 (OLYMPUS, Tokyo Japan) at a resolution of 96 dots per inch (1920 × 1440 pixels). The scale bar = 50 μm. **c–e** Microscope: AX80 (OLYMPUS, Tokyo, Japan), lenses: UPlanApo (OLYMPUS, Tokyo, Japan), a camera: DP70 (OLYMPUS, Tokyo Japan), and a photo system: DP controller 1.2 (OLYMPUS, Tokyo Japan) at a resolution of 72 dots per inch (4080 × 3072 pixels). The scale bar = 1 mm

A 71-year-old man visited our emergency department due to exacerbation of fatigue and dyspnea on exertion that lasted for two weeks. His past medical history was unremarkable, and his family history was not significant for any serious diseases. Chest imaging revealed a large amount of pericardial fluid, a tumor with calcification in the anterior mediastinum extending into the pericardial inner lumen, and bilateral pleural effusion (Fig. 1a–d). Liver dysfunction, which was thought to be due to congestive hepatopathy, was also observed (aspartate aminotransferase 114 IU/L, alanine aminotransferase 84 IU/L). Based on a diagnosis of cardiac tamponade, approximately 700 ml of slightly bloody, cytology-negative (Class III) exudative fluid (lactate dehydrogenase (LDH) 970 IU/L, proteins 5.4 g/dL) was drained, and symptoms of heart failure improved. Liver dysfunction also disappeared. Computed tomography (CT) imaging with contrast media during drainage showed a contrast-enhanced tumor in the anterior mediastinum (Fig. 1e). Spindle or round to oval epithelial cells and irregular cohesive tissue fragments of epithelial and lymphoid cells, which are characteristic of fine-needle aspirates from thymoma, were not observed in the pericardial fluid (Fig. 2a). The patient underwent thoracoscopic tumor biopsy by left-sided access without creating a pleuro-pericardial communication. From macroscopic findings during the biopsy, direct tumor invasion into the pericardium was considered to be the cause of the bloody exudative pericardial fluid, and the tumor had adhered to the left lung. Cytology of the left pleural effusion collected at the time was negative (Class III), but the protein concentration of the effusion was high (LDH 142 U/L, proteins 4.3 g/dL), suggesting pleural invasion as the cause of pleural effusion in addition to heart failure. Pathological examinations of the biopsy specimen revealed spindle-shaped and oval epithelial cells with low-grade dysplasia arranged in pseudo-rosettes or short fascicles. These cells were admixed with few lymphocytes and were negative for neural differentiation markers such as synaptophysin and chromogranin, which are generally positive for neuroendocrine tumors. These findings indicated a type A thymoma (World Health Organization (WHO) classification) (Fig. 2b). WHO classifies all thymomas as malignant neoplasms, but it is difficult to evaluate thymoma-derived epithelial cells by routine cytology. In addition, immunostaining to distinguish between epithelial cells and mesothelial cells was not performed on pericardial and pleural exudates of our patient. Therefore, cytology class III results did not imply the presence or absence of tumor cells. However, because CT images and macroscopic findings during the biopsy strongly suggested pericardial infiltration of the tumor (Masaoka classification; stage III), four courses of chemotherapy with cisplatin, doxorubicin, and methyl-prednisolone were given as preoperative chemotherapy, resulting in a partial response. The pericardial drainage tube inserted for emergency treatment of cardiac tamponade was removed prior to chemotherapy. Five months after the initial diagnosis, the tumor was resected with a part of the brachiocephalic vein, pericardium, and upper lobe of both lungs, since the tumor had invaded the pericardium inner lumen, mediastinal pleura and bilateral lungs on gross appearance during the surgery. Extensive stumps of resected specimens were not analyzed in detail under a microscope. Macroscopically there was no residual tumor, and the resection was considered curative, but not definitive (an R0 or R1 resection). Figure 3a is a dorsal (posterior) view of a formalin-fixed resected specimen showing the tumor extending into the pericardial inner lumen. Although identification of the pericardial structure within the tumor was difficult, the tumor cells showing the same morphology as at the time of biopsy infiltrated into the pericardium near the border with the tumor and the resected specimen was pathologically confirmed to be Masaoka stage III type A thymoma (Fig. 3b–e). It is still unclear whether a good pathological response to chemotherapy leads to a good thymoma prognosis like other thoracic cancers. However, substantial necrosis of the tumor was reported in about half of thymoma patients who received induction treatment [5, 6]. No such effect of chemotherapy was observed in the resected specimen from our patient. Postoperatively, local radiotherapy at a dose of 45 Gy was performed, and no recurrence was observed. However, four years after irradiation, symptomatic heart failure with reduced ejection fraction occurred intermittently and the patient died suddenly due to acute myocardial infarction 7.5 years after the first visit.

## Discussion and conclusions

Small-size mediastinal tumors are usually asymptomatic and often found incidentally on chest CT scans. Since detection of mediastinal tumors on chest X-ray examinations alone is hard, some patients have not been properly diagnosed until a huge tumor has formed. Direct invasion of an advanced mediastinum tumor into the surrounding organs can cause symptoms such as cough, chest pain, and dyspnea, and occasionally induces cardiac tamponade due to pericardial infiltration. Type A thymoma has a better prognosis compared to type B thymoma, which consists of round or polygonal epithelial cells generally with a prominent lymphocytic component [3]. Most of the reported thymomas with cardiac tamponade were type B or type AB (mixed thymoma) [1, 2]. To the best of our knowledge, one case of cardiac tamponade due to thymoma, judged to be type A, was reported after release of the 1999 WHO classification [4]. The subsequent prognosis of the patient is unknown. There were several similar cases prior to 1999 which were later classed as type A thymomas in reviews, although criteria regarding evaluation as type A were unclear [1, 2, 7, 8]. In addition, two autopsy cases of highly suspected type A thymoma were reported as pericardial ectopic thymoma that may progress to cardiac tamponade in 1997 [9, 10]. The prognosis of thymoma with Masaoka stage III–IV or cardiac tamponade is generally poor, but in this case, long-term disease-free survival was achieved by multidisciplinary treatment (preoperative chemotherapy, surgical resection and postoperative local radiotherapy), in accordance with the histological type [3].

The role of postoperative radiotherapy (PORT) in patients with completely resected stage III thymoma is still controversial and the Japanese thymic tumor practice guidelines (the Japan Lung Cancer Society, 2020) do not indicate a recommended level of PORT for these patients. However, recent retrospective studies tend to support PORT. Rimner et al. reported that PORT significantly improved overall survival (OS) of patients with stage II/III disease, even in completely resected cases [11]. Liao et al. also observed a significant OS benefit from PORT in completely resected stage III thymoma [12]. Although standard treatment for stage III type A thymoma with cardiac tamponade has not been established, we chose radiation therapy as a treatment option for our patient. On the other hand, a linear radiation dose–response relationship between the mean absorbed heart dose and the mortality risk due to cardiovascular disease has been reported in cases such as lymphoma and malignant mesothelioma. The risk is highest 10 years after mediastinal radiotherapy and depends on the age of the patient [13]. Moreover, it was also reported that a higher heart dose was a risk factor for cardiovascular disease in long-term survivors of thymoma treated with PORT [12]. Since no symptoms of heart failure were observed before irradiation, irradiation to the heart was probably involved in the heart failure and acute myocardial infarction in our patient. Every effort should be made in the irradiation planning to reduce the heart dose as much as possible.

More than 80% of patients with pericarditis have idiopathic or viral causes, and malignant diseases are rarely the cause. However, in cases with pericardial fluid, 12–23% are attributable to malignant diseases [14]. Typical diseases that cause pericarditis as the initial symptom of occult cancer are lung cancer, non-Hodgkin lymphoma, and myeloid leukemia, but thymoma should also be considered in the differential diagnosis [14]. Pericarditis is a prognostic factor for cancer mortality and neoplastic cardiac tamponade usually develops in patients with highly advanced cancer [14]. Therefore, treatment is often limited to pericardial drainage as a palliative approach, especially in the elderly. However, depending on the histological type, aggressive treatment may be successful, as in this case. Fine needle aspiration cytopathology (FNAC) may be useful for elderly patients for whom it is difficult to perform a surgical biopsy for a mediastinal tumor. Although cytologists require sufficient experience, the utility of FNAC has been reported even for thymoma [15, 16]. Zakowski et al. reported that the accuracy of FNAC diagnosis was 100% in 18 cases of thymoma [16].

In summary, we herein present a 71-year-old male patient with cardiac tamponade due to type A thymoma. In this case, long-term survival was achieved by multidisciplinary treatment. This case indicates that neoplastic cardiac tamponade, even in elderly patients, should not necessarily be regarded as a terminal cancer and requires a systematic investigation for underlying causes.

## Data Availability

Data sharing is not applicable to this article as no datasets were generated or analyzed during the current study.

## Abbreviations

LDH: Lactate dehydrogenase
CT: Computed tomography
WHO: World Health Organization
PORT: Postoperative radiotherapy
OS: Overall survival
FNAC: Fine needle aspiration cytopathology
